# Supplementary material for: Cracking double-blind review: Authorship attribution with deep learning
Source: PLoS One. 2023 Jun 30;18(6):e0287611. doi: 10.1371/journal.pone.0287611 (PMC10313031; doi:10.1371/journal.pone.0287611)
Supplement: Attachment — Submitted filename: Reviewers_response_plos_one_papernet.pdf [file pone.0287611.s001.pdf]

Cracking double-blind review: authorship attribution with deep learning

Author Response Letter to the Editor and the Reviewers

We thank the editor and the reviewers for their helpful and detailed comments, identification of improvement potential, and constructive criticism. We answer all feedback by inserting our response into the provided feedback text in blue.

Kind regards,

Leonard Bauerfeld, Angel Romero, Manasi Muglikar, Davide Scaramuzza.

Editor

Thank you for submitting your manuscript to PLOS ONE. After careful consideration, we feel that it has merit but does not fully meet PLOS ONE's publication criteria as it currently stands. Therefore, we invite you to submit a revised version of the manuscript that addresses the points raised during the review process.

We thank the Editor for the reassuring statement that our work has merit. We have thoroughly revised the manuscript to improve its quality and clarity and addressed all points raised by the editor and the reviewers.

Both reviewers agree the paper has margin to improve, specifically regarding the following points:

1) The behavior of the proposed system when trained on a dataset where each author has fewer than 100 papers.

We thank the editor and the reviewers for this valuable suggestion. We have now conducted two sets of experiments - within the limitations of the available compute - to address this point. In summary, we find that our approach can cope remarkably well with large datasets even when fewer texts are available, as we achieve a 56.4% attribution accuracy with over 5000 authors and *only 25 papers* per author (20 for training, 5 for testing). For details, please see the *new Sec. 5.3* in the manuscript.

In detail, we ran the following two experiments (see Table 5, reproduced below):

1. For a fixed number of 25 papers per author, we have varied the amount of authors up to 5292 candidate authors. We observe that increasing the number of authors decreases the accuracy slightly. However, comparing 63% accuracy for 226 candidate authors to 56.4% accuracy for 5292 candidate authors shows that our system can cope with large numbers of authors even if only little training data is available.

   Training even larger datasets with tens of thousands of authors is not feasible given the memory and compute requirements of such very large experiments. For our largest dataset we train up to two weeks on a server with an RTX 8000 GPU and 1TB memory. Here, it is important to point out that we solve a classification task and thus the memory requirements heavily depend on the number of classes (e.g. authors) that are possible. Thus it is not the same to train with 1000 authors with 100 papers each or 10000 authors with 10 papers each.

Table 5. Results on the trimmed datasets with a decreasing number of papers per author available for training and testing. Even at less than 1/8th of the original data, the network still retains 75% of its performance on the D200-C dataset. When the number of authors is increased 20 times with only 25 papers per author (D50T25-C), the performance drops by a mere 6 percentage points.

Dataset	D200-C					D100T25-C	D50T25-C
Authors	226	226	226	226	226	2070	5000
Papers/Author	>200	200	100	50	25	25	25
Accuracy [%]	81.6	76.6	75.1	71.4	62.5	57.0	56.4

2. For a mid-sized dataset (D200-C) with 226 different authors we have reduced the number of papers per author systematically from 200 to 25 while keeping the number of candidate authors the same (see Table 5). Decreasing the amount of data available per author decreases the accuracy from 81.6% (full dataset, >200 papers per author) to 75.1% (100 papers per author) down to 63% (25 papers per author). Thus with only 1/8th of the data the model still retains 3/4 of its performance. This again supports our claim that our approach performs very well if overall enough data to finetune the transformer+MLP is available.

2) A thorough discussion on the results, in terms of false attribution, correlations, pitfalls, limitations when used in a real-world scenario, etc.

We thank the editor and reviewers for suggesting a more detailed analysis as the analysis we've conducted indeed further strengthens the paper and highlights the quality of the proposed approach. The results are integrated into the paper in the *new section 5.4*.

As a first step, we have analysed the overall attribution accuracy for different authors and provide an overview of the statistics in Fig. 4 (reproduced below). First, we notice that the performance across authors is very similar and the interquartile range on the 'worst case' D100-C dataset is only 30 percentage points, already indicating that the model does not do "all or nothing" (some authors always correct, others never) but performs consistently.

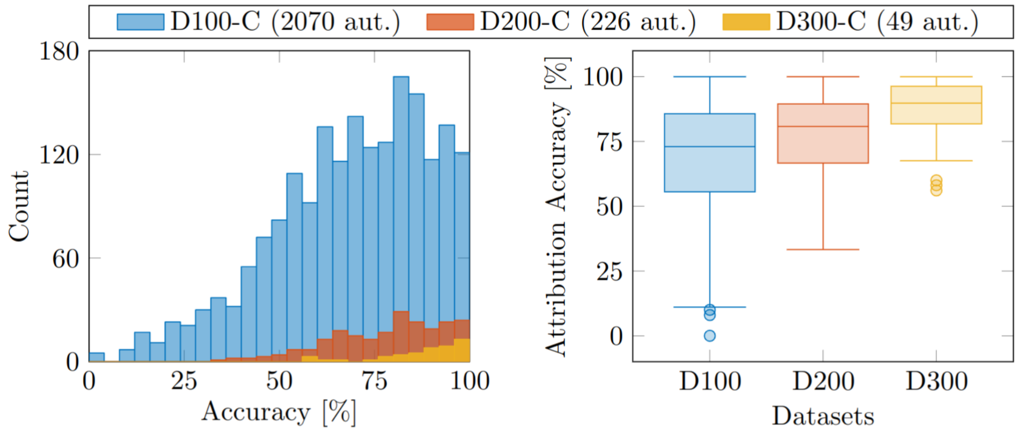

To further analyse what contributed to "easily identifiable" or "hard to identify" authors we find that the primary factor is the number of training samples available. As our datasets are generated based on an author having "at least" a certain number of papers on arXiv, the number of papers per author varies greatly across the datasets. Hence, in a second step we analysed this aspect in greater detail (see Fig. 5, reproduced below).

First we confirm the intuition that more samples per author lead to better attribution accuracy. Secondly we find that, for a given number of training samples per author, having an overall larger dataset helps (meaning, more possible different authors). For 100 papers per author, for example, the D100-C clearly outperforms the D200-C in accuracy, despite the D100-C having 2070 candidate authors and the D200-C only having 226 candidate authors.

This finding confirms our scaling analysis: the method can be expected to scale very well to even larger datasets, given the compute capabilities to actually train it. Already now, our authorship attribution dataset is a factor of 10 to 100 larger than the datasets used in previous work.

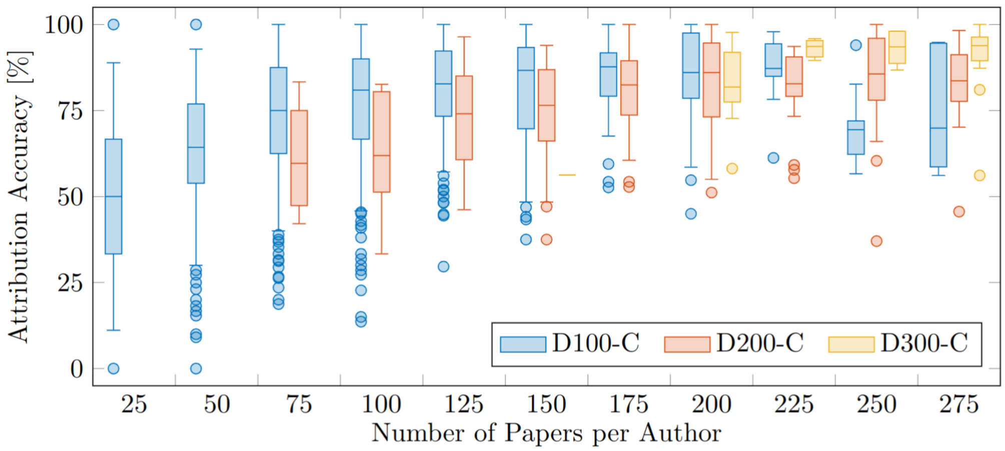

To further understand the capabilities and limitations of our work we analyzed the prediction performance in a multi-author setting (*see newly added Sec. 5.5)*. As a basis for the analysis we use the D100-C dataset on which we achieve an overall accuracy of 73.4%.

1) We find that our approach performs very well on multi-author predictions achieving close to 80% accuracy in predicting one of the authors of multi-author papers.
2) If the task is to correctly predict all of the authors, our model performs well and still achieves close to 50% accuracy, given that the number of authors is known.
3) If the exact number of authors is unknown, but one is only interested in a set of 5 candidate authors that could be authors, in 67% of the cases all candidate authors are among the top-five suggestions of the model.

When the approach is used in the real-world to support plagiarism detection, especially the last case is relevant as it allows further checking of the relevant authors with a more targeted (but possibly less efficient) method. Case (1) is interesting if the goal is to crack a double-blind review process because knowing one of the authors is usually enough to know which group or lab a work comes from.

If the authors could invest their time on those two particular points, I believe the resulting paper will be much stronger and useful overall.

We hope that the editor and reviewers agree that the thoroughly revised experimental section and detailed analysis of the model performance indeed made the paper much stronger. We thank everyone for their very valuable suggestions and comments.

We have also revised the entire manuscript and included the missing datapoint of our method in Table III, which we were unable to obtain previously due to compute limitations.

Reviewer #1

The manuscript introduces the interesting and significant task of author attribution to the large arXiv preprint service. They explain their methodology and results very clearly, and I am confident their results are correct as presented in the main text. My first main suggestion is to make consistent the claims in the abstract with the findings of the paper. The abstract implies that the D100 data set (with about 2000 authors) yields up to 95% correct attribution (when the text reports up to 70%). I understand the operative use of 'up to' makes the claim in the abstract technically true, but it appeared misleading when I got the the presented results.

We thank the reviewer for the encouraging comments about the clarity of the presented results. We also have changed the abstract to make it more clear that we get 73% accuracy in the 2000 author case.

My second main criticism is that the data set choices are limited in a strange way. Why did the authors structure their datasets with so few authors? I understand they choose to segment the datasets by authors with a certain number of papers, which is a fine choice, but why at the most consider authors with at least 100 papers? The choice of even considering D500 which has only a tiny number of authors also seems strange, because of what practical value is choosing among 7 potential authors? This is by far the largest technical weakness of the paper in my opinion, and it very much limits the significance of the findings. The authors need to address this issue, ideally by adding an experiment with almost all authors, or explaining technical limitations which preclude this possibility and suggesting steps to overcome them.

Our choice of datasets (from D100 to D500) is intentionally selected to cover a broad range of scenarios. While the choice of larger author datasets such as D100 is clear, our smaller author dataset D500 is comparable to the previous datasets, for example 3 authors in [20] or 62 authors in [36]. Since most of the baselines were developed on these small-scale datasets, we wanted to present a complete evaluation of our method and also include results on smaller datasets, as discussed in Sec. 3.3. Furthermore, the datasets are structured in such a way that the number of candidate authors is roughly logarithmically spaced.

In response to the concerns regarding an even larger dataset, we have now conducted two sets of experiments - within the limitations of the available compute - to address this point. In summary, we find that our approach can cope remarkably well with large datasets even when fewer texts are available, as we achieve a 56.4% attribution accuracy with over 5000 authors and *only 25 papers* per author (20 for training, 5 for testing). In detail, we ran the following two experiments (see Table 5 reproduced below, and the *new Sec. 5.3*):

1. For a fixed number of 25 papers per author, we have varied the amount of authors up to 5292 candidate authors. We observe that increasing the number of authors decreases the accuracy slightly. However, comparing 63% accuracy for 226 candidate authors to 56.4% accuracy for 5292 candidate authors shows that our system can cope with large numbers of authors even if only little training data is available.

Table 5. Results on the trimmed datasets with a decreasing number of papers per author available for training and testing. Even at less than 1/8th of the original data, the network still retains 75% of its performance on the D200-C dataset. When the number of authors is increased 20 times with only 25 papers per author (D50T25-C), the performance drops by a mere 6 percentage points.

Dataset	D200-C					D100T25-C	D50T25-C
Authors	226	226	226	226	226	2070	5000
Papers/Author	>200	200	100	50	25	25	25
Accuracy [%]	81.6	76.6	75.1	71.4	62.5	57.0	56.4

Training even larger datasets with tens of thousands of authors is not feasible given the memory and compute requirements of such very large experiments. Here, it is important to point out that we solve a classification task and thus the memory requirements heavily depend on the number of classes (e.g. authors) that are possible.

Looking at the scaling in terms of storage and memory requirements, the memory requirement (for the one-hot encoding) scales linearly in the number of classes as well as the number of samples. The same can be observed for the reference histogram embedding. Including more papers in the dataset yields more possible authors that can be cited and thus increases the histogram size. Thus, if we include more authors in the dataset while keeping the number of papers per author constant, our memory requirement scales (at least) quadratically with the number of authors. It is not the same to train with 1000 authors with 100 papers each or 10000 authors with 10 papers each.

This limitation could be overcome by a) using more hardware or b) a custom pytorch implementation which doesn't store sparse embeddings and only 'densifies' them at runtime for the currently processed batch. As this is primarily an engineering challenge, we have not focused on these aspects. Instead, we provide experimental evidence that our method performs very well on larger datasets.

Next to practical issues in terms of compute power, the second issue with including even larger parts of arxiv is author ambiguity. As mentioned in the manuscript, arXiv does not have unique author identifiers that are publicly accessible. Henceforth we have to rely on the authors' name (and the subsequent pre-filtering step, Sec. 3.1.3) to disambiguate authors. This becomes increasingly error-prone with increasing dataset size and thus generating datasets with less than 25 papers per author will likely prove problematic.

2. As an additional ablation study, for a mid-sized dataset (D200-C) with 226 different authors we have reduced the number of papers per author systematically from 200 to 25 while keeping the number of authors the same. Decreasing the amount of data available per author decreases the accuracy from 81.6% (full dataset, >200 papers per author) to 75.1% (100 papers per author) down to 63% (25 papers per author). Thus with only 1/8th of the data the model still retains 3/4 of its performance. This again supports our claim that our approach performs very well if overall enough data to finetune the transformer+MLP is available.

My third main suggestion is that the paper needs a more significant discussion of false attributions, which if this tool is used in some capacity for decision making or plagiarism detection etc, is a critical risk to assess. For example, they report how often they can identify a single author of a paper, but if they try to identify all authors, how many do they get right, and how many are falsely attributed?

We thank the reviewer for suggesting a more detailed analysis as the analysis we've conducted indeed further strengthens the paper and highlights the quality of the proposed approach. The results are integrated into the paper in the *new section 5.4*.

As a first step, we have analysed the overall attribution accuracy for different authors and provide an overview of the statistics in Fig. 4 (reproduced below). First, we notice that the performance across authors is very similar and the interquartile range on the 'worst case' D100-C dataset is only 30 percentage points, already indicating that the model does not do "all or nothing" (e.g. some authors always correct, others never) but performs consistently.

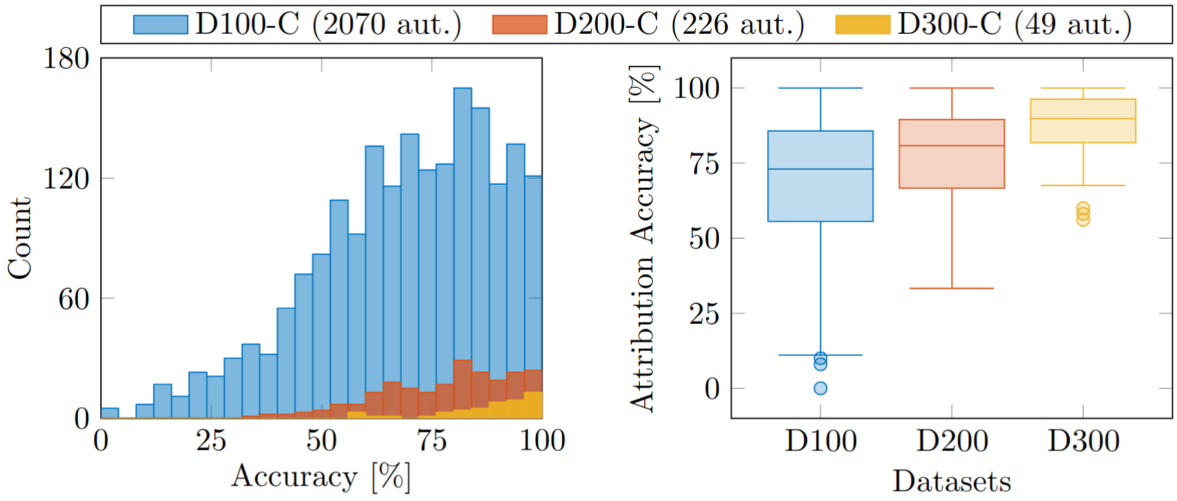

To further analyse what contributed to "easily identifiable" or "hard to identify" authors we find that the primary factor is the number of training samples available. As our datasets are generated based on an author having "at least" a certain number of papers on arXiv, the number of papers per author varies greatly across the datasets. Hence, in a second step we analysed this aspect in greater detail (see Fig. 5, reproduced below).

First we confirm the intuition that more samples per author lead to better attribution accuracy. Secondly we find that, for a given number of training samples per author, having an overall larger dataset helps. For 100 papers per author, for example, the D100-C clearly outperforms the D200-C in accuracy, despite the D100-C having 2070 candidate authors and the D200-C only having 226 candidate authors.

This finding confirms our scaling analysis: the method can be expected to scale very well to even larger datasets, given the compute capabilities to actually train it. Already now, our authorship attribution dataset is a factor of 10 to 100 larger than the datasets used in previous work.

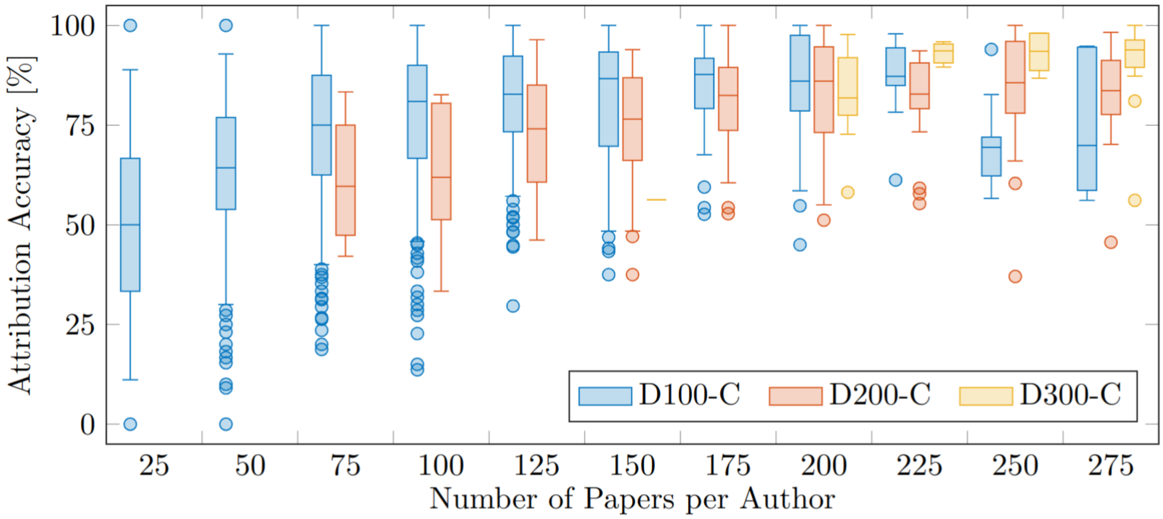

To further understand the capabilities and limitations of our work we analyzed the prediction performance in a multi-author setting (*see newly added Sec. 5.5)* as suggested by the reviewer. As a basis for the analysis we use the D100-C dataset on which we achieve an overall accuracy of 73.4%.

1) We find that our approach performs very well on multi-author predictions achieving close to 80% accuracy in predicting one of the authors of multi-author papers.
2) If the task is to correctly predict all of the authors, our model performs well and still achieves close to 50% accuracy, given that the number of authors is known.
3) If the exact number of authors is unknown, but one is only interested in a set of 5 candidate authors that could be authors, in 67% of the cases all candidate authors are among the top-five suggestions of the model.

When the approach is used in the real-world to support plagiarism detection, this last case is especially relevant as it allows further checking of the relevant authors with a more targeted (but possibly less efficient) method. Case (1) is interesting if the goal is to crack a double-blind review process because knowing one of the authors is usually enough to know which group or lab a work comes from.

Reviewer #2:

The paper proposes a multichannel NN to predict authorship. It is based on a destilbert representation of paper content and a histogram-based representation of cited authors. To train the network, the authors created a public dataset from arxiv. The results are reproduceable, as the code is publicly available, including the code to create the dataset.

The application is interesting, and it constitute in the academic world a real issue. Authors, intentionally or unintentionally, provide many hints about authorship in double-blind submissions. In that regard, the motivation is nice, but the paper was very superficial in regards to the proposition and the experiments.

a) The paper is brief and provides few details about the proposed method. One needs to dig into the code to understand how the text was processed, the references were pre-pared, the details of the NN illustrated in Fig. 2, etc. The availability of the code is very important, but the paper need to be self-contained. There are many choices that need to be further justified. This is necessary in the dataset preparation, and on the NN architecture.

We have addressed this and greatly extended the section that explains the processing of our dataset (Sec. 3.1) to make the paper self-contained. We give a detailed explanation on how the preprocessing works. For the exact regular expressions we refer the reader to the source code. The network architecture itself as well as choice of network architecture (especially w.r.t the reference embedding) is discussed in Sec. 4. and we have added more details to the discussion.

b) The authors chose to train the NN using people with at least 100 papers. While many faculties in the academic career may display such behavior, this is not true for younger researchers, say PH.D Candidates. I miss a discussion on that choice, and on the behavior of the system regarding authors with fewer papers.

We thank the reviewer for this valuable suggestion. We have now conducted two sets of experiments - within the limitations of the available compute - to address these points. In summary, we find that our approach can cope remarkably well with large datasets even when fewer texts are available, as we achieve a 56.4% attribution accuracy with over 5000 authors and *only 25 papers* per author (20 for training, 5 for testing).

In detail, we ran the following two experiments (see Table 5 reproduced below, and the *new Sec. 5.3* in the manuscript):

1. For a fixed number of 25 papers per author, we have varied the amount of authors up to 5292 candidate authors. We observe that increasing the number of authors decreases the accuracy slightly. However, comparing 63% accuracy for 226 candidate authors to 56.4% accuracy for 5292 candidate authors shows that our system can cope with large numbers of authors even if only little training data is available.

Table 5. Results on the trimmed datasets with a decreasing number of papers per author available for training and testing. Even at less than 1/8th of the original data, the network still retains 75% of its performance on the D200-C dataset. When the number of authors is increased 20 times with only 25 papers per author (D50T25-C), the performance drops by a mere 6 percentage points.

Dataset	D200-C					D100T25-C	D50T25-C
Authors	226	226	226	226	226	2070	5000
Papers/Author	>200	200	100	50	25	25	25
Accuracy [%]	81.6	76.6	75.1	71.4	62.5	57.0	56.4

Training even larger datasets with tens of thousands of authors is not feasible given the memory and compute requirements of such very large experiments. For our largest dataset we train up to two weeks on a server with an RTX 8000 GPU and 1TB memory. Here, it is important to point out that we solve a classification task and thus the memory requirements heavily depend on the number of classes (e.g. authors) that are possible. Thus it is not the same to train with 1000 authors with 100 papers each or 10000 authors with 10 papers each.

2. For a mid-sized dataset (D200-C) with 226 different authors we have reduced the number of papers per author systematically from 200 to 25 while keeping the number of authors the same. Decreasing the amount of data available per author decreases the accuracy from 81.6% (full dataset, >200 papers per author) to 75.1% (100 papers per author) down to 63% (25 papers per author). Thus with only 1/8th of the data the model still retains 3/4 of its performance. This again supports our claim that our approach performs very well if overall enough data to finetune the transformer+MLP is available.

c) I miss a discussion about the correct and incorrect predictions. I wonder whether only very well “known” authors are recognized, and what features lead to successful/unsuccessful predictions.

We thank the reviewer for suggesting a more detailed analysis as the analysis we’ve conducted indeed further strengthens the paper and highlights the quality of the proposed approach. The results are integrated into the paper in the *new section 5.4*.

As a first step, we have analysed the overall attribution accuracy for different authors and provide an overview of the statistics in Fig. 4 (reproduced below). First, we notice that the performance across authors is very similar and the interquartile range on the ‘worst case’ D100-C dataset is only 30 percentage points, already indicating that the model does not do “all or nothing” (some authors always correct, others never) but performs consistently.

To further analyse what contributed to “easily identifiable” or “hard to identify” authors we find that the primary factor is the number of training samples available. As our datasets are generated based on an author having “at least” a certain number of papers on arXiv, the number of papers per author varies greatly across the datasets. Hence, in a second step we analysed this aspect in greater detail (see Fig. 5, reproduced below).

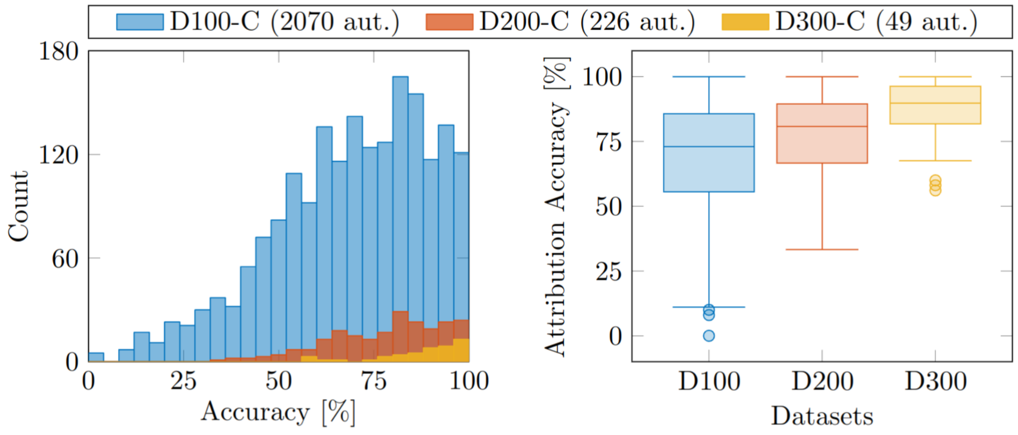

First we confirm the intuition that more samples per author lead to better attribution accuracy. Second, we find that, for a given number of training samples per author, having an overall larger dataset helps. For 100 papers per author, for example, the D100-C clearly outperforms the D200-C in accuracy, despite the D100-C having 2070 candidate authors and the D200-C only having 226 candidate authors.

This finding confirms our scaling analysis: the method can be expected to scale very well to even larger datasets, given the compute capabilities to actually train it. Already now, our authorship attribution dataset is a factor of 10 to 100 larger than the datasets used in previous work.

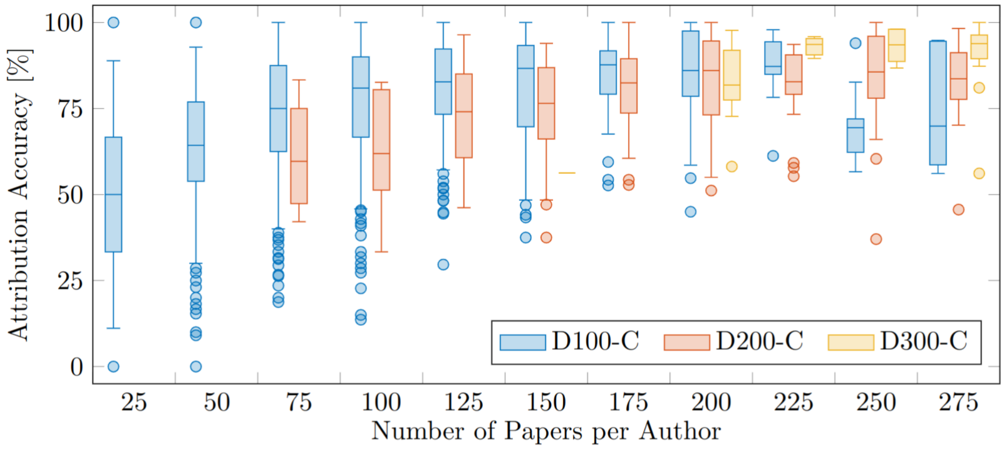

To further understand the capabilities and limitations of our work we analyzed the prediction performance in a multi-author setting (*see newly added Sec. 5.5)*. As a basis for the analysis we use the D100-C dataset on which we achieve an overall accuracy of 73.4%.

1) We find that our approach performs very well on multi-author predictions achieving close to 80% accuracy in predicting one of the authors of multi-author papers.
2) If the task is to correctly predict all of the authors, our model performs well and still achieves close to 50% accuracy, given that the number of authors is known.
3) If the exact number of authors is unknown, but one is only interested in a set of 5 candidate authors that could be authors, in 67% of the cases all candidate authors are among the top-five suggestions of the model.

When the approach is used in the real-world to support plagiarism detection, this last case is especially relevant as it allows further checking of the relevant authors with a more targeted (but possibly less efficient) method. Case (1) is interesting if the goal is to crack a double-blind review process because knowing one of the authors is usually enough to know which group or lab a work comes from.

d) The authors claim in the introduction that they provide insights on how to identify authorship, but these insights are somewhat obvious, except perhaps for some “style” of writing introduction/abstract. Again, a more detailed discussion on that needs to be provided.

We agree with the reviewer insofar as the insights provided seem “obvious”. However, even proving the obvious with actual data has its scientific merit. We would like to gain a deeper understanding of what information or text parts the neural network uses to identify an author. However, it is difficult to pinpoint this information for large transformer architectures and likely a future research direction. We have released our code and believe that this is a stepping stone for the community facilitating future research in this direction.

In times where machine learning tools are becoming more powerful and widely adopted, the ability to identify papers can adversely affect the double-blind review process. We have adapted the wording in the introduction and clarified our discussion to highlight that we limit ourselves to simple mitigation strategies (e.g. limiting the number of self citations) as we believe this is a viable attempt to mitigate the risk.
